# Is Adolescence a Window of Opportunity for Prepregnancy Obesity Prevention?

**DOI:** 10.1111/ppe.70037

**Published:** 2025-06-09

**Authors:** Romy Gaillard

**Affiliations:** ^1^ The Generation R Study Group, Erasmus University Medical Center Rotterdam the Netherlands; ^2^ Department of Pediatrics, Sophia Children's Hospital Erasmus University Medical Center Rotterdam the Netherlands

Maternal prepregnancy obesity and excessive weight gain in pregnancy remain highly prevalent worldwide and are major risk factors for maternal and fetal pregnancy complications [1]. A large‐scale individual participant data meta‐analysis using data from 29 Western birth cohorts showed that a third of women with a normal BMI at the start of their pregnancy experienced maternal or fetal pregnancy complications, whereas the percentage of any pregnancy complication ranged from 50% to 61% for women with obesity grade I to obesity grade III [1]. The percentages of complications were highest among those women who also gained a high amount of weight in their pregnancy, corresponding to a weight gain > 16 kg, but the effects of excessive gestational weight gain on the risks of pregnancy complications were lower than the effects of maternal prepregnancy obesity [1]. The adverse impact of maternal prepregnancy obesity and excessive gestational weight gain is not limited to the perinatal period, but rather adversely affects maternal and offspring health across the life course [2]. For example, in offspring, especially maternal prepregnancy obesity is associated with substantially higher risks of obesity and an adverse cardiovascular profile from early childhood onwards, and increased risks of premature mortality and hospital admissions from cardiovascular events in adulthood [2, 3, 4].

Despite an overwhelming body of evidence linking suboptimal maternal weight before and during pregnancy to health complications for both mother and offspring, developing and implementing successful, scalable strategies to optimise maternal weight remains a major challenge. Lifestyle intervention studies using diet and exercise interventions in pregnant women with obesity have not shown strong beneficial effects on pregnancy outcomes or long‐term maternal or offspring outcomes [5]. Often, these intervention studies targeted one single lifestyle factor, focused mainly on diet or exercise as intervention targets, suffered from low compliance to lifestyle advice, and mainly started after the critical preconception period [5]. Identification of novel, modifiable intervention targets and their critical periods to optimise maternal weight status, even before pregnancy, is urgently needed.

In this issue of *Paediatric and Perinatal Epidemiology*, Mason and colleagues [6] report the findings from the Life‐course Experiences And Pregnancy (LEAP) project, a study performed among 607 women who participated since adolescence in a prospective cohort study of weight‐related health (Project EAT). The authors assessed whether adolescent risk factors with links to adult overweight or obesity are also associated with higher risks of prepregnancy obesity and high gestational weight gain. Adolescent risk factors were prospectively ascertained between the ages of 11 and 18 years. Pregnancy‐weight measures were self‐reported by questionnaire. On average, the participants' first live birth occurred 8 years before the study questionnaire. The majority of women were white, had a middle to high socio‐economic status and educational level, and a mean age of 27.6 years at first childbirth. Approximately 17% of women had prepregnancy obesity, and 24% gained excessive gestational weight according to the Institute of Medicine Guidelines. The authors showed that adolescent overweight or obesity, body dissatisfaction, weight‐related teasing, binge eating, and unhealthy weight control behaviours were consistently associated with a higher risk of prepregnancy obesity, whereas healthy home food availability was associated with a lower risk. The effects remained after adjustment for socio‐economic factors. Weaker effects of these risk factors with excessive gestational weight gain were present. No effects for food insufficiency, regular family meals, chronic dieting, or depressive symptoms were present [6]. The novelty of this study lies in its focus on a unique critical period—adolescence—rather than the period just before or during pregnancy, and the focus on a broad range of risk factors beyond weight, diet, and exercise, including eating behaviours, mental health factors, and food environment factors.

An important limitation of the study is the use of self‐reported pregnancy weight measures, which are prone to reporting bias. This bias may be especially pronounced in this cohort due to the study's focus on weight and eating behaviours, and the long gap between pregnancy and the data collection by questionnaire. To address this issue, the authors collected internal validation pregnancy weight data based on medical records in a small subsample of women, conducted a quantitative bias analysis, and performed misclassification‐adjusted analyses, which affected almost all associations between adolescent risk factors and pregnancy‐weight‐related outcomes [6]. These analyses clearly highlight the importance of striving towards weight data obtained from medical records or by actual study measurements to prevent the detection of spurious associations when possible. Furthermore, the study suffered from high attrition rates, increasing the risk of selection bias and affecting the generalisability of the observed results, especially to less affluent populations. Finally, causality cannot be established from this observational study, and some of the identified risk factors may not be causal factors per se, but rather be proxy measurements for other underlying causal factors.

Nevertheless, this study adds to the observational evidence that adolescence may be a critical period, where unhealthy weight, lifestyle, eating behaviours, and mental health problems originate or further accumulate, leading to higher risks of prepregnancy obesity among couples wishing to conceive [7]. As offspring of more birth cohorts worldwide are now reaching adulthood, further studies exploring the influence of adolescent risk factors on prepregnancy obesity and excessive gestational weight gain are necessary to replicate findings and explore effects across more diverse populations. Ideally, these studies should use repeated, prospective measurements of risk factors throughout adolescence into adulthood to identify risk factor trajectories that may lead to prepregnancy obesity in couples wishing to conceive and higher risks of excessive gestational weight gain. These studies should not only focus on women, but rather on couples and consider the influence of relationships, the partner, and their potential risk factors on maternal risk factor trajectories and obesity risk.

Interventions in the adolescent period require a population‐level approach and are of interest from a public health perspective [7, 8]. Intervention studies in the critical adolescent period, which focus on optimising health behaviours and weight, that also explain health benefits for future offspring and Developmental Origins of Health and Disease (DOHAD) concepts are emerging [8, 9, 10]. These small studies suggest some improved understanding of DOHAD concepts and motivation for improving health behaviours in adolescents and young adults, but their impact on actual long‐term health behaviours and weight status remains to be established [9, 10]. Well‐designed multi‐disciplinary intervention studies, which target diverse risk factors and integrate DOHAD concepts, can provide important insights into how to achieve optimal weight and health behaviour trajectories in both girls and boys from adolescence to adulthood, and their potential to reduce maternal and offspring health complications across the life course.

## Author Contributions

The author takes full responsibility for this article.

## Conflicts of Interest

The author declares no conflicts of interest.

## Data Availability

The author has nothing to report.
